# Quantifying the Responses of Three *Bacillus cereus* Strains in Isothermal Conditions and During Spray Drying of Different Carrier Agents

**DOI:** 10.3389/fmicb.2018.01113

**Published:** 2018-05-29

**Authors:** Verônica O. Alvarenga, Fernanda B. Campagnollo, Arthur K. R. Pia, Deborah A. Conceição, Yuri Abud, Celso Sant’Anna, Miriam D. Hubinger, Anderson S. Sant’Ana

**Affiliations:** ^1^Department of Food Science, Faculty of Food Engineering, University of Campinas, Campinas, Brazil; ^2^Laboratory of Biotechnology (Labio), Metrology Applied to Life Science Division – National Institute of Metrology, Quality and Technology (Inmetro), Duque de Caxias, Brazil; ^3^Department of Food Engineering, Faculty of Food Engineering, University of Campinas, Campinas, Brazil

**Keywords:** spores, low water activity, thermal processing, dried foods, sporeforming bacteria, dairy products, milk, foodborne pathogens

## Abstract

Spray drying is a widely used method for producing milk powder. This process is not aimed to cause microbial inactivation, thus sporeforming bacteria may be abundant in the microbiota of milk powder. The first aim of this study was to determine the inactivation kinetics parameters in capillary tubes of three *Bacillus cereus* strains (436, B63, 540) in three menstrua (whole milk, phosphate buffer, and talc suspension) at 90, 100, and 110°C. *D*-values for *B. cereus* in the three menstrua were not significantly different at the highest tested temperature (*p* > 0.05). Thus, talc was chosen as a carrier agent to allow the recovery of *B. cereus* from spray dried materials given its low interference on inactivation kinetics. *B. cereus* spores were also inoculated in whole milk and skim milk following spray drying at 95, 105, and 110°C (outlet temperature). After the spray drying runs, *B. cereus* spores were counted and the number of decimal reductions (γ) calculated. A correlation between the small diameter of the particles with the survival of spores of three *B. cereus* strains was found, and *B. cereus* 436 presented consistently the lowest γ no matter temperature and a carrier agent. The highest γ was found when talc powder was used, which suggest that this carrier agent does not protect *B. cereus* spores during spray drying. Spray drying of milk can lead to up to 4 γ (strain 540) of *B. cereus* spores but depending on the strain less than one γ (strain 436) could be observed. This study contributes to the knowledge on the microbiology of low water activity foods by providing novel findings regarding the fate of three *B. cereus* strains to different spray drying conditions. Acknowledging the variability of inactivation of *B. cereus* during spray drying is key in the current context of food safety in which the quantification of effects of unit operations must be known for the validation of processes and development of more robust formulations.

## Introduction

Milk is an important source of proteins with high biological value, vitamins, and minerals (Pereira, 2014). Besides the relevant role in human nutrition, it is also important from the economic point of view (Zoccal, 2016). Nonetheless, the complex microbiota present in milk may lead to its spoilage or association with foodborne diseases (Machado et al., 2017). Because of these issues, preservation strategies such as drying have been employed by dairy industry to extend milk’s shelf-life.

Drying of milk is used to reduce loss associated with microbiological spoilage, to extend shelf-life and to facilitate commercialization as well as transportation. Milk powder is a shelf-stable product that can be used as an ingredient in chocolate, dairy desserts and several other food formulations (Cook and Johnson, 2009). The drying process of milk can be carried out by different methods, such as, drum dryer, roller dryer, bed dryer. However, spray drying is likely the most used method for milk drying (Birchal and Passos, 2005). Spray drying is one of the most efficient and most economical water reduction methods employed by the food industry (Sollohub and Cal, 2010). During the spray drying process, inlet air and outlet temperatures may reach from 200 to 250°C and about 100°C, respectively (Anandharamakrishnan and Ishwarya, 2015). When exposed to these conditions for a short time (20–30 s), microorganisms could be injured (Bhandari et al., 2008). Even though spray drying is not applied with the purpose of causing microbial inactivation, the temperature employed in the process may result in microbial death (Huang et al., 2017). Sporeforming bacteria, however, are expected to survive to some extent and dominate the microbiota of dairy powder products (Becker et al., 1994; IDF, 2016; Yuan et al., 2018). Because of that, some industries establish a maximum limit of 100 CFU/g of *B. cereus* group in dried milk intended for industrial applications, such as for formulation of infant formulas (Becker et al., 1994).

In fact, the presence of pathogenic and spoilage sporeforming bacteria in milk powder and milk powder-based products has been reported in the literature (LiCari, 1970; in’t Veld et al., 1993; Becker et al., 1994; Reyes et al., 2007; Beuchat et al., 2013; Stoeckel et al., 2013). Among these, *Bacillus cereus* is a highly relevant bacterium due to its high prevalence (Banykó and Vyletelová, 2009; Kumari and Sarkar, 2014) and association with outbreaks linked to dairy products (Becker et al., 1994; Beuchat et al., 2013). Although sporeforming bacteria, such as *B. cereus*, are not dominant in the microbiota of raw milk (Neubeck et al., 2015; Kable et al., 2016), the thermal and mechanical procedures employed throughout processing can lead to a higher abundance of sporeforming bacteria in milk powder (Becker et al., 1994; Kumari and Sarkar, 2016). Even though the low water activity of powder does not allow microbial growth, survival of vegetative cells for long periods can be observed (Spanu, 2016). Spores will remain latent and withstand low a_w_ conditions for even much more time in dried products. Despite this, when milk powder is rehydrated, spores can find suitable conditions for germination and outgrowth, likely resulting in spoilage or foodborne diseases (McHugh et al., 2017). Given the above, it becomes evident that the contamination of milk powder with sporeforming bacteria may have several implications for the microbiological safety and stability of formulated foods (McHugh et al., 2017). Thus, in addition to guaranteeing the use of high-quality raw materials (Lang et al., 2017), the quantification of the effect of unit operations applied during milk powder production is vital in the contemporary framework of food safety. However, to the best of author’s knowledge, no data exist concerning the fate of *B. cereus* spores yielded to different conditions of spray drying. This study first aimed to estimate the inactivation kinetic parameters in capillary tubes of three *B. cereus* strains in three agents (whole milk, phosphate buffer, and talc suspension). Then, the fate of three strains of *B. cereus* during spray drying processes held at different temperature conditions and three agents (whole milk, skim milk and talc powder) was also studied.

## Materials and Methods

### Strains and Preparation of Spore Suspensions

Three strains of *B. cereus* were used in this study: 436 (isolated from chocolate), strain B63 (ready-to-eat meal), and strain 540 (dairy product). These strains are deposited in the *Bacillus* culture collection of the Fundação Oswaldo Cruz (Rio de Janeiro, Brazil). These *B. cereus* strains were selected for this study as they presented different resistances to the spray drying process in previous tests (data not shown).

Spore suspensions were prepared according to the methodology described by Pflug (1999) in nutrient broth (Kasvi, Curitiba, Brazil) supplemented with manganese sulfate (10 ppm) (Synth, Diadema, Brazil). Suspensions were incubated at 30°C for 30 days. Sporulation was daily monitored with malachite green staining. Once, spores corresponded to more than 90% of microscopic observation, suspensions were harvested with sterile distilled water followed by centrifugation (1500 × *g* for 20 min at 4°C) (Sorvall Legend XTR Thermo fisher, Waltham, MI, United States). Then they were resuspended in sterile distilled water and stored at -20°C until use.

### Preparation and Inoculation of Carrier Agents

Different menstrua (also referred as “carrier agents” for spray dryer experiments) were used: (a) whole milk [3.5% (v/v) fat], phosphate buffered saline (PBS) pH 7.2 and talc suspension (10 g/L), for the determination of inactivation kinetics in capillary tubes and (b) talc suspension, whole milk, and skim milk [0.0% (v/v) fat] for the fate experiments to spray drying processes. The lots of whole and skim milk used in the experiments were tested regarding the presence of *B. cereus* according to Bennett et al. (2015) and showed counts of <10 spores/g (data not presented).

Monobasic sodium phosphate [2.44 g/L] (Dinâmica, Diadema, Brazil), dibasic sodium phosphate [8.09 g/L] (Dinâmica, Diadema, Brazil), and sodium chloride [4.25 g/L] (Dinâmica, Diadema, Brazil) were used for PBS preparation, and pH was adjusted to 7.2 (Taylor and Pérez, 2015). Talc (Dinâmica, Diadema, Brazil) was resuspended in distilled water [10 g/L] (Kloepper, 1981). PBS and talc suspension were sterilized at 121°C for 15 min.

Spores were inoculated into 50 mL of menstruum/carrier agent with a final concentration of 7.5 ± 0.5 log_10_ spores/g. The initial count of each suspension was confirmed after thermal shock at 80°C for 30 min (Bennett et al., 2015), followed by decimal dilutions in peptone water 0.1%, plating onto MYP (Mannitol Egg Yolk Polymyxin) agar (Acumedia, Lansing, MI, United States), and incubation at 30°C for 48 h.

### Inactivation and Fate Experiments of *B. cereus*

The inactivation of *B. cereus* strains studied first in capillary tubes, then in spray dryer. Capillary tubes were chosen for use in the inactivation kinetics experiments taking into account the high temperatures employed in the spray drying process (>150°C and >95°C for inlet air and outlet temperatures, respectively) and due to their the isothermal conditions (Pflug, 1999). Thus, these experiments were carried out in controlled oil bath described in the next section.

### Inactivation of *B. cereus* in Capillary Tubes

The capillary tubes (ø external 1.5 mm; ø internal 1.1 mm; length 75 mm, Lamiglass, São Paulo, Brazil) were filled with 25 μL of each mestrumm inoculated with *B. cereus* spores. After that, they were sealed with a blowtorch. The sealed tubes were heated in an oil bath (Cole-Parmer Polystat, model 12101-20, Vernon Hills, IL United States) at the following conditions: 90°C for 150 min, 100°C for 15 min, and 110°C for 1.5 min. A total of 27 experiments was carried out in duplicate (*n* = 54), it was used 3 strains, 3 menstrua, and 3 temperature conditions. At different times (*n* = 6 points/temperature), time zero was included. For each temperature was used a regular interval of time. After heating, ten capillary tubes were collected and immediately immersed in a cold-water solution containing detergents. After 2 min in the ice bath, the capillary tubes were sanitized with 70% (v/v) ethanol solution and opened under aseptic conditions. Further, the contents of the capillary tubes (200 μL in total for each data point) were transferred to a new tube containing 1.8 mL sterile peptone water 0.1% (w/v), and 10-fold dilutions were prepared using sterile peptone water 0.1%. Then, they were plated onto MYP agar (Acumedia, Lansing, MI, United States), following incubation at 30°C for 48 h. Characteristic colonies of *B. cereus* were counted as proposed by Bennett et al. (2015). The counts of *B. cereus* were expressed as spores per grams. The inactivation kinetic parameters were determined after fitting the log-linear model using Equation 1 in Ginafit (Geeraerd et al., 2005) add-in for Microsoft^®^ Excel (Microsoft, Redmond, United States). *D*-values were determined at studied temperatures. The *D*-values were calculated for constant environmental conditions.

(1) $$\log\;N\;(t)\;=\;\log\;N_{0}\;-\;\frac{t}{D}$$

Where *N*_0_, initial counts of spores count in the menstruum (spores/g); *N*, spores count after heating in the menstruum (spores/g); *t*, heating time; *D*, time required for decimal reduction of the spore population.

Then, the estimated *D*-values for each strain, at each temperature, and menstruum were used to determine the thermal coefficient (*z*-value) using linear regression by log_10_ *D*-value versus temperature (°C) in Microsoft Excel^®^ (Microsoft, Redmond, WA, United States).

### Fate of *B. cereus* During Spray Drying Processes

The experiments were conducted in a spray dryer model SD 1.0 (LabMaq, Ribeirão Preto, Brazil). Three inlet air temperatures were tested: 190 ± 2, 170 ± 2, and 150 ± 2°C, which resulted in the following outlet temperatures: 110 ± 5, 105 ± 5, and 95 ± 5°C, respectively. Temperatures were selected considering the industrial applications and limitation of the equipment (maximum inlet air temperature ∼200°C). The other drying parameters were fixed: drying air flow (84 m^3^/h), feeding flow (7 × 10^-4^ m^3^/h), compressed air flow (2.4 m^3^/h), and compressed air pressure (0.25 MPa). The carrier agents inoculated with spore suspensions (500 g at a concentration of 7.5 ± 0.5 log_10_ spores/g of dry weight) were spray dried in separate experiments. In total, 27 drying experiments were conducted (3 strains × 3 carrier agents × 3 temperatures), with two independent repetitions (*n* = 54). Additionally, for each tested condition, control experiments were run using the non-inoculated carrier agents. No sporeforming bacteria were recovered from the dried control samples.

After each drying process, the spray dryer (drying chamber, atomizing nozzles, cyclone, pipes, and exhaustion duct) was washed, sanitized, and sterilized. All spray dryer components were immersed in a 1.0% (v/v) solution of alkaline detergent Divostar Quattro^®^ (Diversey, São Paulo, Brazil) for 30 min at 30°C and rinsed with water. Then, they were immersed in a 0.5% (v/v) solution of acid detergent Divostar Pascal^®^ (Diversey, São Paulo, Brazil) for 20 min at 30°C, and then rinsed with water again. For sanitation, the equipment was immersed in a 0.25% (v/v) solution of peracetic acid Divostar Forte^®^ (Diversey, São Paulo, Brazil) for 20 min, rinsed with water, and sterilized at 121°C for 40 min. Other security measures adopted were: installation of a HEPA type filter with a porosity of 1 μm (LabMaq, Riberão Preto, Brazil) in the spray dryer’s exhaustion outlet and the allocation of the spray dryer inside a protected room. The room was cleaned and disinfected with benzalkonium chloride 10% (v/v) prior and after each spray drying process.

The concentration of *B. cereus* spores in each carrier agent was determined before and after the drying processes. Counting was carried out following the methodology described by Bennett et al. (2015) using MYP agar (Acumedia, Lansing, MI, United States) and incubation at 30°C for 48 h. Before the drying process, 10 g of inoculated carrier agent was sampled and exposed to heat shock activation treatment. After the spray drying process, 1 g of powder was resuspended in 9 mL of diluent. For milk, sodium citrate (Dinâmica, Diadema, Brazil) was used, whereas for talc suspension, peptone water 0.1% was the diluent employed. The heat shock conditions applied before and after the spray drying processes were different: at 80°C for 30 min and 75°C for 20 min for counts of *N*_0_ and *N*, respectively. The heat shock conditions were chosen based on preliminary tests conducted using several time/temperature combinations available in the literature (Smelt et al., 2008): 90°C for 10 min, 80°C for 30 min, 70°C for 20 min and 75°C for 20 min (data not shown).

The number of decimal reductions (γ) of *B. cereus* spores was determined for the experiments run with three strains in three different carrier agents and three drying conditions. The calculation of γ used the concentration of sample as spores per grams of dry weight, before and after the drying process, as shown in Equation 2.

(2) $$\gamma \;=\;log\left(\frac{N_{0}}{N}\right)$$

Where *N*_0_, initial counts of spores by dry weight of carrier agent (spores/g dry weight); *N*, spores count after the spray drying process by dry weight of the sample (spores/g dry weight).

Also, the lethal effects of inlet and outlet air temperatures on the survival of *B. cereus* spores were studied. A pseudo *z*-value, i.e., the temperature change required for one-log reduction of γ of each drying process, was estimated as proposed by LiCari (1970) and Kim and Bhowmik (1990). This model (Equation 3) proposes a linear relationship between γ and the outlet temperature of the drying air.

(3) γ = a(T)+b

Where *N*_0_, initial count of spores by dry weight of carrier agent (spores/g of dry weight); N, spores count after the spray drying process by dry weight of carrier agent (spores/g of dry weight); a, slope coefficient; and b, linear coefficient.

### Determination of Water Activity and Moisture Content of Dried Powders

Water activity and moisture content were determined for all carrier agents, before and after the drying process. Water activity was determined in a water activity measuring device (Aqualab, 4TEV, Decagon Devices, Pullman, WA, United States) at 25°C. The moisture content of the samples was determined by infrared scale (Gehaka IV3100, São Paulo, Brazil) (Arslan et al., 2015). The measurements were performed in triplicate.

### Effect of Particle Size Distribution on the Survival of *B. cereus* Spores in Spray Drying Processes

Particle size distribution was determined by laser diffraction in Mastersize 2000 (Malvern, Worcestershire, United Kingdom) equipped with wet sample unit (Hydro 2000s). Ethyl alcohol 99.5% (Dinâmica, Diadema, Brazil) was used as a carrier agent for particle dispersion. The average diameter of the particles was determined based on the diameter of the same-volume sphere, De Brouckere diameter D_4,3_ presented in Equation 4 (Mugele and Evans, 1951). The analyses were performed in triplicate. After that, the correlation between the particle diameter and the survival of spores of three *B. cereus* strains in the three carrier agents was tested.

(4) $$D_{4,3}\;=\;\frac{\sum_{i=1}^{n}n.d_{i}^{4}}{\sum_{i=1}^{n}n.d_{i}^{3}}$$

Where *d*_i_, diameter of the particles; n, number of particles.

### Scanning Electron Microscopy (SEM) of Different Carrier Agents

The powders were mounted onto SEM stubs using double-sided carbon tape, coated with 5 nm platinum, and examined in an FEI Quanta FEG 450 scanning electron microscope, operating at an accelerating voltage of 10–15 kV (Brienzo et al., 2016).

### Statistical Analyses

Differences in the number of decimal reductions (γ) and *D* and *z*-values calculated for the different strains were compared by an analysis of variance (ANOVA, *post hoc* analysis Tukey test with a critical value *p* = 0.05; and *t*-test *p* = 0.05). For the pseudo *z*-value, the differences were tested by a Wil Cox test *p* = 0.05). Pearson correlation (*p* = 0.05) was tested using R (version 3.3.1) (*The R Foundation for Statistical Computing*, Vienna, Austria).

## Results

### Determination of Thermal Resistance of *B. cereus* Strains in Capillary Tubes

**FIGURE 1:**
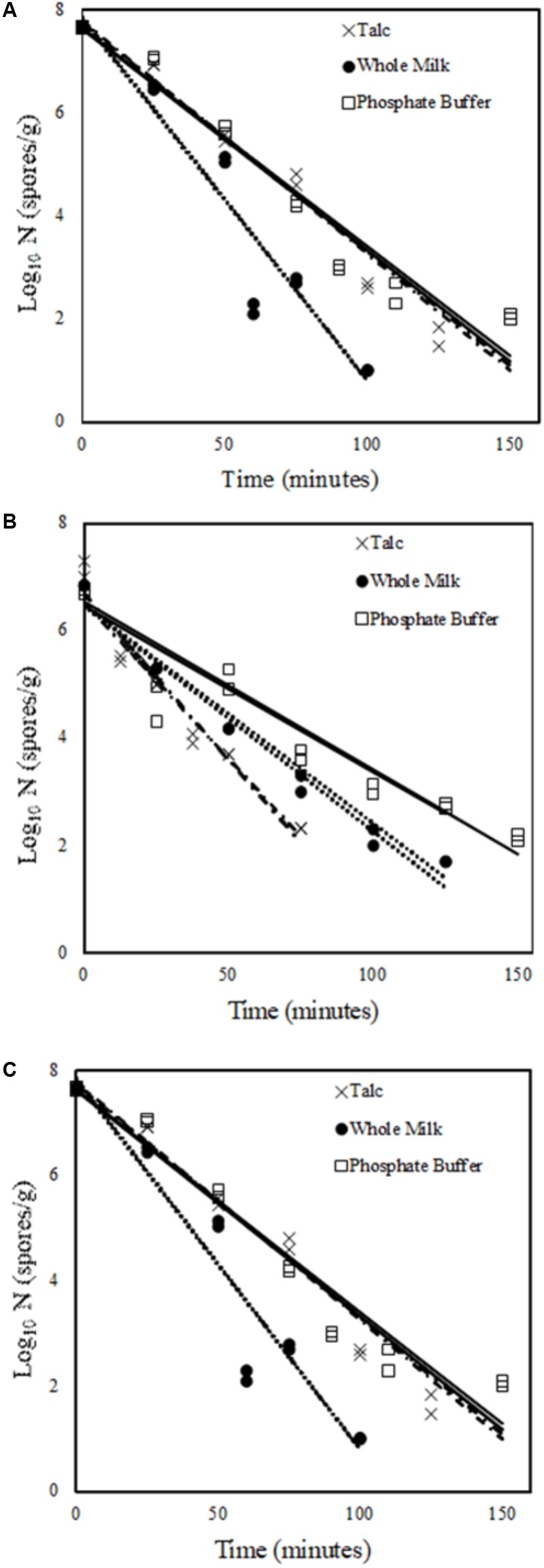
Inactivation curve of *Bacillus cereus* spores at 90°C, **(A)** strain 540, **(B)** strain B63, **(C)** strain 436 in three different menstrua (×) Talc Suspension; (•) Whole Milk; (□) Phosphate buffer solution pH 7.2 (PBS).

**Figure 1** shows inactivation curves of spores of three *B. cereus* strains (436, B63, and 540). The curves followed a first-order inactivation kinetics (*R*^2^ > 0.95) in capillary tubes in the three different menstrua.

The *D*-values determined for *B. cereus* strains studied in the different menstrua and temperatures are presented in **Table 1**. The *D*-values were influenced by strain and carrier agent when considered specific temperatures (**Table 1**). For instance, *B. cereus* B63 presented the highest *D*_90°C_ values in whole milk and PBS (*p* > 0.05). *B. cereus* B63 showed a *D*_90°C_ value of 35.64 min in PBS and 24.31 min whole milk (*p* < 0.05), while *B. cereus* 436 showed the highest *D*_90°C_ values in PBS, respectively (**Table 1**). The highest *D*_110°C_ values for *B. cereus* were obtained in talc suspension and PBS (*p* < 0.05). At 110°C, the effect of menstruum was only evident for *B. cereus* 436, for which the highest *D*-value was obtained in PBS (**Table 1**) (*p* < 0.05). The water activity of the menstrua studied was significantly different (talc suspension, 0.998 ± 0.002; PBS, 0.992 ± 0.001; and whole milk, 0.995 ± 0.002) (p < 0.05).

**Table 1:** *D*-values for different strains of *Bacillus cereus* inoculated in different menstruum in capillary tubes.

Strain	Temperature (°C)	*D*-value (min)		
		Menstruum		
		Talc Suspension	PBS	Whole milk
**540**	**90**	10.72 ± 0.35	17.71 ± 0.00	20.06 ± 1.20
	**100**	4.56 ± 0.06	4.31 ± 0.30	3.87 ± 0.05
	**110**	0.49 ± 0.01	0.62 ± 0.04	0.25 ± 0.01
**B63**	**90**	17.08 ± 0.90	35.64 ± 3.90	24.31 ± 1.81
	**100**	2.81 ± 0.05	2.30 ± 0.10	3.09 ± 0.03
	**110**	0.32 ± 0.01	0.67 ± 0.01	0.26 ± 0.01
**436**	**90**	23.03 ± 0.00	23.03 ± 0.00	14.39 ± 0.00
	**100**	1.75 ± 0.09	1.48 ± 0.13	1.91 ± 0.01
	**110**	0.20 ± 0.01	0.30 ± 0.02	0.19 ± 0.01

**FIGURE 2:**
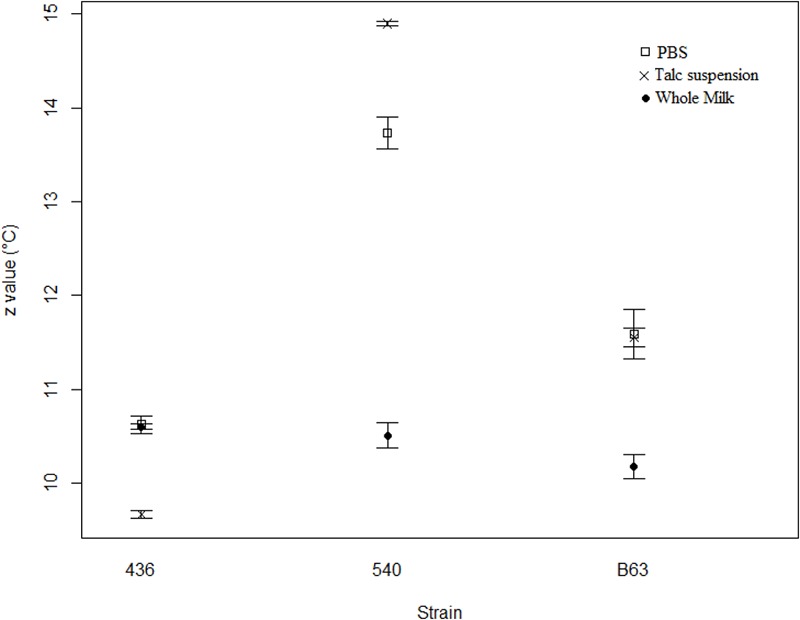
*z*-values for *Bacillus cereus* spores in different heating media (•) whole milk; (□) PBS, (×) talc suspension.

**Figure 2** shows the *z-*values found for the three *B. cereus* strains in the three menstrua. The *z*-values of *B. cereus* 436, *B. cereus* 540 and *B. cereus* B63 varied from 9.7 to 10.6°C, 10.5 to 14.9°C and from 10.2 to 11.6°C, respectively. The lowest *z*-values for the *B. cereus* strains were obtained in whole milk (*p* < 0.05). On the other hand, significantly higher *z*-values were obtained in talc suspension and PBS compared to the values obtained in whole milk (*p* < 0.05). A comparison indicated no significant differences between the *z*-values obtained in talc suspension and PBS (*p* > 0.05).

### Fate of *B. cereus* During the Spray Drying Processes

**Table 2** shows the number of decimal reductions (γ) during the spray drying processes varied with strain and carrier agent studied. The highest γ for most of *B. cereus* strains were observed when talc powder was used as a carrier agent, except for two conditions, for *B. cereus* 540 at inlet temperature 190°C. When talc powder was the carrier agent, no effect of outlet temperature on the γ of the three *B. cereus* strains studied was observed (*p* > 0.05) (**Table 2**). When whole milk was used as a carrier agent, the γ did not vary significantly for *B. cereus* B63 and 436 (*p* > 0.05), whereas when skim milk was employed, only the γ of *B. cereus* B63 was not significantly different at outlet temperature conditions studied (*p* > 0.05).

**Table 2:** Comparison of different spray drying process on *Bacillus cereus* log reduction (γ).

Strain	Inlet temperature (°C)	Outlet temperature (°C)	Carrier agent		
			Talc	Whole milk	Skim Milk
**540**	150	95	3.09 ± 0.03^A^	2.26 ± 0.12^B^	2.19 ± 0.05^B^
	170	105	3.19 ± 0.05^A^	2.65 ± 0.07^B^	2.21 ± 0.05^B^
	190	110	3.33 ± 0.09^B^	4.41 ± 0.25^A^	4.08 ± 0.19^A^
**B63**	150	95	3.17 ± 0.23^A^	2.19 ± 0.13^B^	2.45 ± 0.09^B^
	170	105	3.18 ± 0.07^A^	2.34 ± 0.10^B^	2.52 ± 0.14^B^
	190	110	3.27 ± 0.19^A^	2.40 ± 0.01^B^	2.76 ± 0.08^B^
**436**	150	95	1.62 ± 0.06^A^	0.75 ± 0.08^B^	0.06 ± 0.01^B^
	170	105	1.67 ± 0.08^A^	0.89 ± 0.16^B^	1.01 ± 0.05^B^
	190	110	1.69 ± 0.09^A^	1.18 ± 0.10^B^	1.39 ± 0.03^B^

*^*A,B*^Different letters in the same line indicates significant difference (*p* < 0.05) by Tukey test for same strain in different carrier agent.*

As shown in **Table 2**, *B. cereus* 436 was the most resistant strain to spray drying processes (*p* < 0.05), with γ varying from 0.06 log_10_ spores/g of dry weight in skim milk (95°C outlet temperature) to 1.69 log_10_ spores/g of dry weight in talc powder. On the other hand, *B. cereus* 540 showed more than 4 log_10_ spores/g of dry weight being observed in spray drying processes of whole and skim milk (110°C outlet temperature) being the most sensitive to these conditions (**Table 2**). The γ of *B. cereus* B63 showed less variability no matter temperature and carrier agent (2.19–3.27 log_10_ spores/g of dry weight) (**Table 2**).

The drying conditions significantly affected the pseudo *z*-values (*p* < 0.05 – **Table 3**). The pseudo *z-*values calculated for the three *B. cereus* strains were significantly higher in talc powder compared with whole and skim milk (*p* < 0.05). *B. cereus* B63 presented the highest pseudo *z*-values in whole milk and skim milk (*p* > 0.05). The lowest pseudo *z*-values were found for *B. cereus* 540 (in whole and skim milk) and 436 (in skim milk) (*p* > 0.05). The *z* (capillary tube) and pseudo *z-*values obtained considering the outlet air drying temperature were significantly different (*p* < 0.05) (analysis not presented).

**Table 3:** Pseudo *z*^1^-values for the assessed drying conditions.

Strain	Pseudo *z*-value (°C)		
	Talc	Whole milk	Skim milk
**540**	67.5^c^	7.8^e^	9.2^e^
**B63**	163.9^b^	69.6^c^	53.5^c^
**436**	211.2^a^	38.0^d^	11.2^e^

*^*1*^Thermal coefficient (°C) determined by linear regression in function of the number of reductions (γ) occurred in the drying process. ^*a*-*h*^Different letters indicate significant difference (*p* < 0.05) by WilCox test.*

### Characteristics of the Powders Obtained at Different Drying Conditions

The powder’s characteristics, moisture contents, and the water activity are shown in **Table 4**. The moisture content was influenced by air drying temperature (*p* < 0.05). The initial moisture content, before drying, was 2.60 g/100 g wet basis for talc suspension, 6.60 g/100 g wet basis for skim milk and 10.60 g/100 g for whole milk. The water activity was 0.998 (Talc suspension), 0.994 (skim milk), and 0.995 (whole milk).

**Table 4:** Solid content and water activity of final powder with different carrier agents in different conditions of spray drying.

Carrier agent	Temperature (^o^C)	Water activity	Solid content
			(g/100 sample
			g wet basis)
**Talc**	150	0.543 ± 0.007^a^	99.30 ± 0.13^a^
	170	0.539 ± 0.008^a^	99.54 ± 0.26^a^
	190	0.490 ± 0.005^b^	99.89 ± 0.13^a^
**Whole milk**	150	0.448 ± 0.025^c^	92.71 ± 0.93^e^
	170	0.444 ± 0.037^c^	94.27 ± 0.10^d^
	190	0.429 ± 0.011^d^	94.68 ± 0.32^d^
**Skim milk**	150	0.423 ± 0.013^e^	92.38 ± 0.44^g^
	170	0.399 ± 0.030^e^	92.74 ± 0.15^g^
	190	0.367 ± 0.007^f^	93.47 ± 0.39^f^

*^*a*-*g*^Different letters in the same column present significant difference by *t*-test (*p* < 0.05).*

The powders produced in higher outlet drying temperature showed higher moisture content and lowest aw. The moisture content ranged between 94 and 92 g/100 g of wet basis; between 93 and 92 g/100 g of wet basis; and 99 g/100 g wet basis in whole milk, skim milk and talc powder, respectively. Water activity ranged from 0.45 to 0.43 (whole milk); 0.42 to 0.37 (skim milk); and 0.54 to 0.49 (talc powder).

### Effect of Particle Size Distribution on the Survival of *B. cereus* Spores in Spray Drying Processes

**FIGURE 3:**
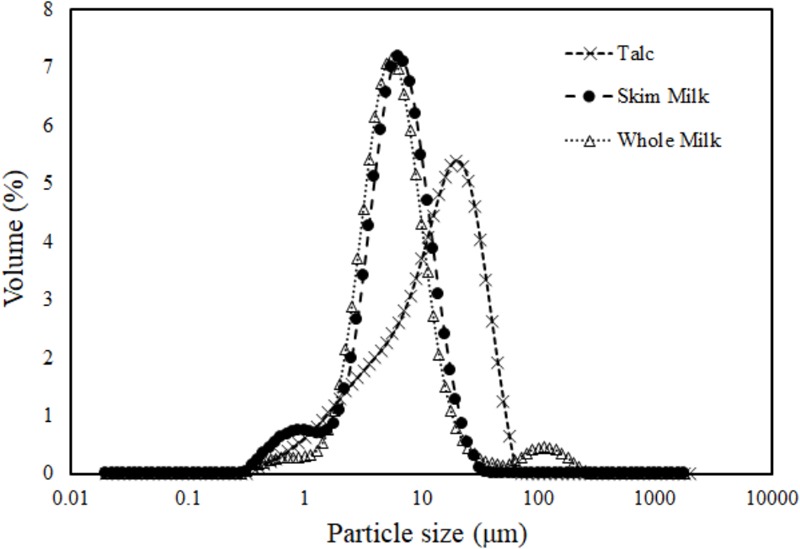
Particle size distribution 190°C (inlet temperature) for different carrier agent: of talc (×), whole milk (△), skim milk (•).

The particle diameter ranged from 6.42 to 7.66 μm, 9.03 to 10.51 μm, and 14.74 to 15.74 μm for skim milk, whole milk, and talc powder, respectively (**Table 5**). The particle size distribution for whole and skim milk was very close, differing from the distribution observed for talc powder (**Figure 3**).

**Table 5:** Particle characteristics after different spray drying process.

Carrier agent	Inlet temperature (^o^C)	D_[4.3]_ (μm)^1^	d_10_ (μm)^2^	d_50_ (μm)^3^	d_90_ (μm)^4^	Span^5^
**Talc**	150	15.34 ± 0.43	2.29 ± 0.04	13.13 ± 0.45	31.93 ± 0.80	2.26 ± 0.02
	170	14.74 ± 0.41	2.11 ± 0.10	12.10 ± 0.33	31.62 ± 0.81	2.44 ± 0.01
	190	14.95 ± 0.49	2.14 ± 0.12	12.46 ± 0.86	31.63 ± 0.04	2.37 ± 0.17
**Whole milk**	150	9.03 ± 0.29	2.79 ± 0.05	5.71 ± 0.06	11.75 ± 0.10	1.57 ± 0.02
	170	10.29 ± 0.78	2.55 ± 0.15	5.72 ± 0.21	15.50 ± 0.73	2.27 ± 0.02
	190	10.51 ± 0.95	2.70 ± 0.18	6.17 ± 0.55	14.80 ± 2.20	1.96 ± 0.16
**Skim milk**	150	6.42 ± 0.09	2.08 ± 0.08	4.97 ± 0.01	11.15 ± 0.23	1.83 ± 0.07
	170	7.66 ± 0.90	1.97 ± 0.18	5.92 ± 0.22	12.77 ± 0.49	1.83 ± 0.01
	190	7.61 ± 0.00	2.24 ± 0.04	6.49 ± 0.08	14.33 ± 0.30	1.86 ± 0.08

*^*1*^D_*[4,3]*_, average particle diameter; ^*2*^d_*10*_, distribution of diameter of up to 10% of the particles; ^*3*^d_*50*_, distribution of diameter of up to 50% of the particles; ^*4*^d_*90*_, distribution of diameter of up to 90% of the particles; ^*5*^span, distribution size determined by (d_*90*_ – d_*10*_)/(d_*50*_).*

**FIGURE 4:**
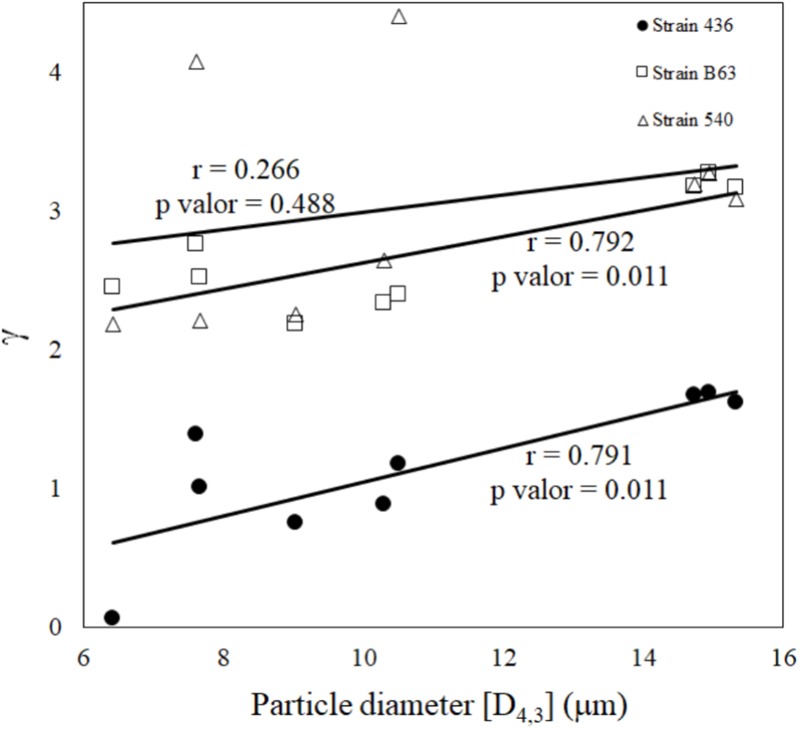
Pearson correlation between particle size and logarithmic survival ratio of *Bacillus cereus* spores during the spray drying. (□) strain 540, (△) strain B63, and (•) strain 436.

According to **Figure 4**, it can be seen that a reduction in the particle size was correlated with an increase of *B. cereus* 436 and B63 spore’s survival (*r* = 0.79) (*p* < 0.05). However, for *B. cereus* 540, this effect has not been observed under tested conditions (**Figure 4**).

### Scanning Electron Microscopy (SEM) of Different Carrier Agents

**FIGURE 5:**
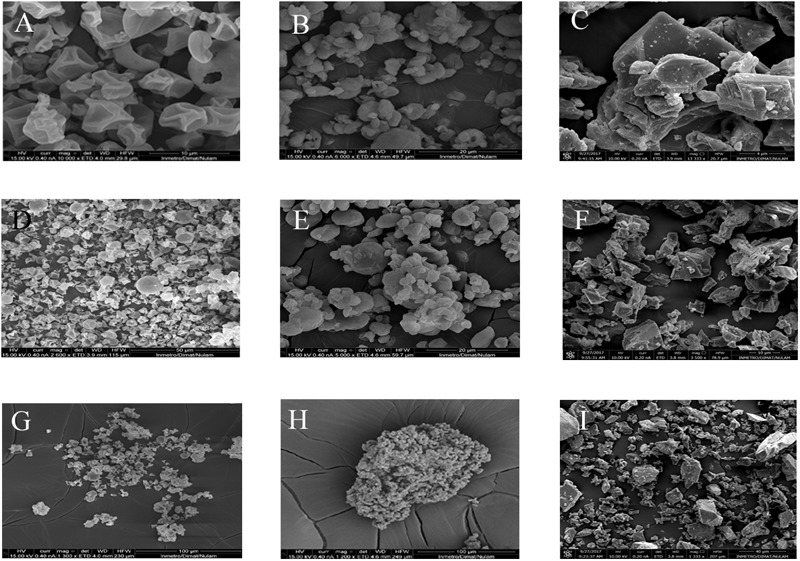
The scanning electron microscopy (SEM) images of microparticles resulting from spray drying process: whole milk **(A,D,G)** skim Milk; **(B,E,H)** and talc powder **(C,F,I)** talc powder. These images illustrate the differences among whole milk, skim milk and talc powder. Talc powder microparticles showed irregular layers with fractures and few agglomerates, while (whole and skim) milk showed an agglomerate structure composed of heterogeneous spheres or polyhedron.

Different morphologies of carrier agents can be observed after the drying processes (**Figure 5**). Talc powder microparticles showed irregular layers with fractures and few agglomerates, while (whole and skim) milk showed an agglomerate structure composed of heterogeneous spheres or polyhedron.

## Discussion

In this study, three menstrua were used to assess the inactivation of spores of different *B. cereus* strains in capillary tubes: PBS, whole milk and talc suspension. *B. cereus* 540, B63 and 436 showed *D*_90°C_ values in capillary tubes within the range described in the literature for milk and dairy products, i.e., between 1.1 and 20.7 min (Mazas et al., 1999a; Stoeckel et al., 2014). As depicted in **Figure 1**, the inactivation curves showed a first-order kinetic pattern likely because the temperature range studied (90–110°C) was close to values in which *D* of *B. cereus* are very low (<1 min) (Mazas et al., 1999a). A trend to the linearization of microbial inactivation curves with the increase of temperature has been reported in the literature (Nolasco Júnior and Massaguer, 2007; Sant’Ana et al., 2009), corroborating our findings.

As shown in **Table 1**, differences in the *D*-values were dependent upon the carrier agent as well as the strain in most of the temperature conditions studied. The results obtained show that the influence of the medium compounds regarding the presence of carbohydrates, proteins, and fat has over the survival of microorganisms (Mazas et al., 1999a; Santillana Farakos et al., 2014). The differences depicted in **Table 1** were more evident when heat resistance of *B. cereus* was determined at 90 and 100°C than at 110°C (*p* < 0.05) (**Table 1**). However, at 110°C the *D*-values were not significantly different among strains and menstrua (*p* > 0.05), except for *B. cereus* 436 (**Table 1**). Given this trend for *D*-values to become non-significant different (*p* > 0.05) at the highest temperature tested, talc suspension was chosen as menstruum to allow the recovery of *B. cereus* spores after spray drying processes, which are typically conducted at very high temperatures (>170°C). This choice is also based on the fact that talc suspension would present no interference on *B. cereus* inactivation kinetics such as food components (fat, proteins, carbohydrates, etc.). Talc is an “odorless white to grayish-white, non-flammable, non-combustible, and non-toxic finely powdered native hydrous magnesium silicate” Anonymous (2018). That has been used as a carrier agent in several segments due to its chemical inertness preventing any chemical or physical reactions (Wypych, 2000). Thus, it can provide an information only of the effect of drying temperature over spore’s survival.

**Figure 2** shows the temperature effects over *B cereus* heat resistance in capillary tubes. A major pattern was found, whole milk *z*-value variation was greater than talc suspension and PBS *z*- values (*p* < 0.05). Heat resistance estimative also may suffer great influence of others physical chemical factors, such as pH (Gaillard et al., 1998; Smelt and Brul, 2014; den Besten et al., 2017). These factors may enhance microbial inactivation quicker with temperature changes (Gaillard et al., 1998), reducing *z*-values (Smelt and Brul, 2014). Since, whole milk average pH was lower than average pH of PBS and talc suspension (pH = 6.7, 7.2 and 8.8, respectively). Thus, variations of up to 5°C and of up to 1.5°C affected *z*-values (among and between strains) in addition to menstruum effect.

The fate of *B. cereus* spores during spray drying of milk was studied because this is a widespread technology used for milk powder production (Schuck, 2002). However, it is known that fat content, solid content, among other factors, affect the inactivation of *B. cereus* during milk thermal processing (Mazas et al., 1999b). Therefore, the effects of milk composition were also taken into account in this study through the evaluation of the fate of *B. cereus* during spray drying of this material. Data in **Table 2** indicate that the variability regarding the heat inactivation of *B. cereus* strains in capillary tubes (**Table 1**) was also observed during spray drying processes. *B. cereus* 436 presented consistently lower γ in comparison with strains 540 and B63 no matter what temperature and carrier agent (talc suspension, whole milk, and skim milk) were considered. This variability among *B. cereus* strains could be related to different water content at the center of the spores of the *B. cereus* strains studied. It is recognized that spores with lower water content have higher resistance to dry heat processes (Tiburski et al., 2014). In fact, microbial inactivation during and after the drying process is known to be associated with thermal, osmotic, oxidative, and desiccation effects (Huang et al., 2017). Nonetheless, as spores are extremely dehydrated structures with their genetic material protected by a crystalline structure (Abhyankar et al., 2011), the temperature could be considered the primary factor is leading to inactivation of spores in suspensions such as tested in this study. During the spray drying process, spore inactivation may occur at different stages. Spores may face drastic changes happened in the carrier agent and suffered from dehydration and thermal effects (Schutyser et al., 2012). The use of carrier agents with different glass transition temperature (T_g_) may impact directly the formation of glassy filaments which would enhance dry heat transfer (Schutyser et al., 2012), and consequently, spore inactivation. The range of *B. cereus* spores T_g_ was described between 50 and 52°C (Sapru and Labuza, 1993). In this study, *B. cereus* spores faced temperatures over their T_g_. In talc suspensions, T_g_ is assumed to be around 60°C (Tri et al., 2013), while in milk T_g_ is over 92°C (Schutyser et al., 2012). Temperatures above T_g_ would result in inactivation of spores (Sapru and Labuza, 1993), enzymes (Schebor et al., 1996) and probiotics (Huang et al., 2017).

The highest γ was obtained when talc powder was used as carrier agent (**Table 2**). These results indicate that talc is a less protective carrier agent for *B. cereus* spores exposed to spray drying. Heat resistance may induce several metabolic pathways according to the composition of the carrier agent employed (Smelt and Brul, 2014). Suspensions with high concentration of minerals may alter pH and water activity shifting the electrolytic balance of the cell leading to changes in membrane composition, extrude protons, protect macromolecules, alter metabolic pathways and favor alkalization (Smelt and Brul, 2014). These suspensions are less protective than suspensions rich in sugars and fats (Smelt and Brul, 2014). These findings are also related to the characteristics of the powders of the three carrier agents tested, i.e., talc suspension, whole and skim milk. The comparison of three carrier agent aimed to explain how *B. cereus* spores are affected by spray drying process (**Table 2**). The use of talc suspension in spray drying experiments would serve a similar purpose as the use of PBS in isothermal conditions. Talc allow the recover of a powder with survivors to spray dryer while it does not interfere or protect the spores from the deleterious effects observed during spray drying.

As shown in **Figures 3–5** and **Table 5**, the particle size diameter was significantly different among these materials (*p* < 0.05). A trend indicates that the protection of *B. cereus* spores increases in smaller particles resulting in a reduction in γ (**Tables 2, 5** and **Figures 3, 4**).

**Figure 5** shows samples of different carrier agent which differ in size and shape. Whole milk particle shows spherical shape, while skim milk particle shows a structure with pores, which might indicate exposure to inflation/deflation process. Both may act as protective structures for spores of *B. cereus*. Milk particle would act as a protective layer keeping spores within the particle. On the other hand, talc particle shows flat structure which may lead spores to greater exposure during spray-drying process (Pispan et al., 2013). Consequently, *B. cereus* spore survival may be much more affected when in talc suspension. Furthermore, it becomes much more visual the difference among the particle size.

Also, a solids concentration of the carrier agent may be determinant to influence droplets size and distribution. Finally, the viscosity is another factor that may affect droplet size because it requires more energy for the atomization and subsequent particle formation (Koca et al., 2015). Milk shows twice the estimated viscosity of aqueous suspension (Deysher et al., 1944). Therefore, it is expected that milk droplets should be smaller due to lactose presence which increases the viscosity (Fu and Chen, 2011). Moreover, droplets with smaller sizes produced due to high-pressure atomization may lead to higher residence time within the drying chamber, which may explain *B. cereus* survival in milk particles (**Figure 5**) (Fu and Chen, 2011).

The correlation between survival and particle diameter was observed in the inactivation of *Salmonella* in milk (LiCari, 1970). The results described by LiCari (1970) showed the opposite pattern described in this work. They found that the smaller the diameter of the particles the greater spore inactivation. In **Figure 4** it can be seen that larger particles were associated with greater *B. cereus* spore inactivation. It is reported that particles with a lower diameter exhibit a tendency to decelerate inside the drying chamber, which results in exposure of the droplet to longer drying time, which consequently may cause greater microbial inactivation (Kim and Bhowmik, 1990; Pispan et al., 2013). However, recently, there were some suggestions that the particle’s morphology, material structure, and position of the microorganism within the carrier agent may influence microbial inactivation (Wang et al., 2016). This influence may be related to the transition phase of drying material to the anamorph phase or film formation (Huang et al., 2017). For the three strains of *B. cereus* evaluated, the lowest numbers of decimal reductions were observed in whole and skim milk, except for the processes at 190°C for strain 540 which presented γ = 4. Some studies suggest that carbohydrates are among the most protective carrier agents in drying processes (Schutyser et al., 2012). The lactose of milk may be enough to form protective layers that may enhance survival of microorganisms (Alves et al., 2016).

Spores protected by solids are two or three times more resistant than spores exposed directly to dry heat (Bruch, 1964). Spore resistance is also affected by the composition of the carrier agents. At this point, mechanisms such as forced convection trigger moisture removal. It can enhance thermal exchange between the drying air and the material to be dried. Later, during the residence time of the droplet within the drying chamber, inactivation can also occur when there is an increase in the chamber temperature (Huang et al., 2017). Thus, LiCari (1970) and Kim and Bhowmik (1990) proposed a parameter, called pseudo *z*-value to evaluate the influence of drying air temperature on the survival of microorganisms. This parameter establishes a linear relationship between the temperature and the log cycle reductions. This correlation can be made because microbial inactivation can be described by a first-order kinetic reaction (Pflug, 1999). Although the outlet temperature does not reflect the thermal history of the droplet, it can be used as an indicator of the survival control of microorganisms in drying processes (Xueyong et al., 2008). At this final stage of drying, the moisture content of the particle is below the critical one, and the moisture removal rate decreases gradually over time. Therefore, during this stage, the temperature of the drop reaches the outlet air temperature, since the drop surface is no longer saturated to maintain the temperature at the corresponding wet bulb temperature, which is a phenomenon limited by diffusion (Bhandari et al., 2008). The findings of this study highlight that the *z*-values obtained in capillary tubes should not be extrapolated to processes where microbial inactivation may be associated with combined mechanisms of heat and of water activity reduction. This assumption is supported by the fact that the mechanisms of heat transfer are different for each system (Berendsen et al., 2015). This difference in *z*-values was also observed between continuous flow heating and capillary heating processes (Berendsen et al., 2015).

## Conclusion

There are few studies that describe the survival of pathogenic microorganisms yielded to the spray drying processes (LiCari, 1970; in’t Veld et al., 1993). Also, the mechanisms involved in the survival of pathogenic microorganisms exposed to the drying process are still poorly understood. In this study, the effects of spray drying conditions on *B. cereus* spores of three strains inoculated in talc solution, whole and skim milk were quantified.

Based on the results of this work, it is not feasible to extrapolate the inactivation kinetic parameters obtained in different heating systems, i.e., capillary tubes and spray dryer. *B. cereus* 436 was generally the most resistant strain to the spray drying processes of milk. The results also indicated that talc powder could be used as a carrier agent in studies aiming to understand the pure effects of the spray drying processes and the responses of *B. cereus* (and likely other sporeforming bacteria) to spray drying processes.

Spray drying of milk can lead to up to 4 γ (strain 540) of *B. cereus* spores but depending on the strain less than one γ (strain 436) could be observed. These results indicate high variability in inactivation of *B. cereus* during spray drying. Acknowledging the variability of inactivation of *B. cereus* during spray drying is key in the current context of food safety in which the quantification of effects of unit operations must be known for the validation of processes and development of more robust formulations. The understanding and quantification of the effects of unit operations employed for the production of ingredients and formulated foods are vital to ensuring shelf-stable and safe products.

## Author Contributions

VA and AS design the study, performed the literature review of spray drying process and spore survival, and performed the data analysis. YA and CS performed the SEM experiments. AP carried out the thermal resistance tests in capillary tubes. FC and MH contributed to data interpretation and literature review and revises the manuscript. All authors have critically reviewed the manuscript and approved the final version.

## Conflict of Interest Statement

The authors declare that the research was conducted in the absence of any commercial or financial relationships that could be construed as a potential conflict of interest.

## References

[B1] Abhyankar W. Pandey R. Ter Beek A. Brul S. de Koning L. J. de Koster C. G. (2011). Reinforcement of *Bacillus subtilis* spores by cross-linking of outer coat proteins during maturation. *Food Microbiol.* 45 54–62. 10.1128/AEM.05031-11 25481062

[B2] Alves N. N. Messaoud G. Ben Desobry S. Costa J. M. C. Rodrigues S. (2016). Effect of drying technique and feed flow rate on bacterial survival and physicochemical properties of a non-dairy fermented probiotic juice powder. *J. Food Eng.* 189 45–54. 10.1016/j.jfoodeng.2016.05.023

[B3] Anandharamakrishnan C. Ishwarya P. (2015). “Introduction to spray drying,” in *Spray Drying Techniques for Food Ingredient Encapsulation* eds Anandharamakrishnan C. Ishwarya P. (Chicago, IL: Willey). 10.1002/9781118863985

[B4] Anonymous (2018). *PubChem Compound Database, CID = 16211421. Natl. Cent. Biotechonolgy Informations*. Available at: https://pubchem.ncbi.nlm.nih.gov/compound/talc#section=Top [Accessed January 15, 2018].

[B5] Arslan S. Erbas M. Tontul I. Topuz A. (2015). Microencapsulation of probiotic *Saccharomyces cerevisiae* var. *boulardii* with different wall materials by spray drying. *LWT - Food Sci. Technol.* 63 685–690. 10.1016/j.lwt.2015.03.034

[B6] Banykó J. Vyletelová M. (2009). Determining the source of *Bacillus cereus* and *Bacillus licheniformis* isolated from raw milk, pasteurized milk and yoghurt. *Lett. Appl. Microbiol.* 48 318–323. 10.1111/j.1472-765X.2008.02526.x 19187503

[B7] Becker H. Schaller G. von Wiese W. Terplan G. (1994). *Bacillus cereus* in infant foods and dried milk products. *Int. J. Food Microbiol.* 23 1–15. 10.1016/0168-1605(94)90218-6 7811567

[B8] Bennett R. W. Tallent S. M. Hait J. M. (2015). “*Bacillus cereus* and Bacillus cereus toxins,” in *Compendium of Methods for the Microbiological Examination of Foods* eds Salfinger Y. Tortorello M. L. (Washington, DC: American Public Health Association) 10.2105/MBEF.0222.036

[B9] Berendsen E. M. Zwietering M. H. Kuipers O. P. Wells-Bennik M. H. (2015). Two distinct groups within the *Bacillus subtilis* group display significantly different spore heat resistance properties. *Food Microbiol.* 45 18–25. 10.1016/j.fm.2014.04.009 25481058

[B10] Beuchat L. R. Komitopoulou E. Beckers H. Bett R. P. Bourdichon F. Fanning S. (2013). Low–water activity foods: increased concern as vehicles of foodborne pathogens. *J. Food Prot.* 76 150–172. 10.4315/0362-028X.JFP-12-211 23317872

[B11] Bhandari B. R. Patel K. C. Chen X. D. (2008). “Spray drying of food materials – process and product characteristics,” in *Drying Technologies in Food Processing* eds Chen X. D. Mujumdar A. S. (West Sussex: Blackwell Publishing) 113–159.

[B12] Birchal V. S. Passos M. L. (2005). Modeling and simulation of milk emulsion drying in spray dryers. *Braz. J. Chem. Eng.* 22 293–302. 10.1590/S0104-66322005000200018

[B13] Brienzo M. Abud Y. Ferreira S. Corrales R. C. N. R. Ferreira-Leitão V. S. de Souza W. (2016). Characterization of anatomy, lignin distribution, and response to pretreatments of sugarcane culm node and internode. *Ind. Crops Prod.* 84 305–313. 10.1016/j.indcrop.2016.01.039

[B14] Bruch C. W. (1964). Some biological and physical factors in dry heat sterilization: a general review. *Life Sci. Space Res.* 2 357–371. 11883444

[B15] Cook F. K. Johnson B. L. (2009). “Microbiological spoilage of cereal products,” in *Compendium of the Microbiological Spoilage of Foods and Beverages* eds Sperber W. H. Doyle M. P. (New York, NY: Springer New York) 223–244. 10.1007/978-1-4419-0826-1_8

[B16] den Besten H. M. W. Aryani D. C. Metselaar K. I. Zwietering M. H. (2017). Microbial variability in growth and heat resistance of a pathogen and a spoiler: all variabilities are equal but some are more equal than others. *Int. J. Food Microbiol.* 240 24–31. 10.1016/j.ijfoodmicro.2016.04.025 27207811

[B17] Deysher E. F. Webb B. H. Holm G. E. (1944). The viscosity of evaporated milks of different solids concentration. *J. Dairy Sci.* 27 345–355. 10.3168/jds.S0022-0302(44)92608-1

[B18] Fu N. Chen X. D. (2011). Towards a maximal cell survival in convective thermal drying processes. *Food Res. Int.* 44 1127–1149. 10.1016/j.foodres.2011.03.053

[B19] Gaillard S. Leguerinel I. Mafart P. (1998). Model for combined effects of temperature, pH and water activity on thermal inactivation of *Bacillus cereus* spores. *J. Food Sci.* 63 887–889. 10.1111/j.1365-2621.1998.tb17920.x

[B20] Geeraerd A. H. Valdramidis V. P. Van Impe J. F. (2005). GInaFiT, a freeware tool to assess non-log-linear microbial survivor curves. *Int. J. Food Microbiol.* 102 95–105. 10.1016/j.ijfoodmicro.2004.11.038 15893399

[B21] Huang S. Vignolles M. Chen X. D. Le Loir Y. Jan G. Schuck P. (2017). Spray drying of probiotics and other food-grade bacteria: a review. *Trends Food Sci. Technol.* 63 1–17. 10.1016/j.tifs.2017.02.007

[B22] IDF (2016). *Bacillus cereus in Milk and Dairy Products. IDF Factsheet.* Available at: http://www.fil-idf.org/wp-content/uploads/2016/12/Bacillus-cereus-in-Milk-and-Dairy-Products.pdf

[B23] in’t Veld P. Soentoro P. Notermans S. (1993). Properties of spores in reference materials prepared from artificially contaminated spray dried milk. *Int. J. Food Microbiol.* 20 23–36. 10.1016/0168-1605(93)90057-N 8251303

[B24] Kable M. E. Srisengfa Y. Laird M. Zaragoza J. Mcleod J. Heidenreich J. (2016). The core and seasonal microbiota of raw bovine milk in tanker trucks and the impact of transfer to a milk processing facility. *MBio* 7 1–13. 10.1128/mBio.00836-16 27555305 PMC4999540

[B25] Kim S. S. Bhowmik S. R. (1990). Survival of lactic acid bacteria during spray drying of plain yogurt. *J. Food Sci.* 55 1008–1010. 10.1111/j.1365-2621.1990.tb01585.x

[B26] Kloepper J. W. (1981). Development of a powder formulation of rhizobacteria for inoculation of potato seed pieces. *Phytopathology* 71:590 10.1094/Phyto-71-590

[B27] Koca N. Erbay Z. Kaymak-Ertekin F. (2015). Effects of spray-drying conditions on the chemical, physical, and sensory properties of cheese powder. *J. Dairy Sci.* 98 2934–2943. 10.3168/jds.2014-9111 25771045

[B28] Kumari S. Sarkar P. K. (2014). Prevalence and characterization of *Bacillus cereus* group from various marketed dairy products in India. *Dairy Sci. Technol.* 94 483–497. 10.1007/s13594-014-0174-5

[B29] Kumari S. Sarkar P. K. (2016). *Bacillus cereus* hazard and control in industrial dairy processing environment. *Food Control* 69 20–29. 10.1016/j.foodcont.2016.04.012

[B30] Lang E. Chemlal L. Molin P. Guyot S. Alvarez-Martin P. Perrier-Cornet J.-M. (2017). Modeling the heat inactivation of foodborne pathogens in milk powder: high relevance of the substrate water activity. *Food Res. Int.* 99 577–585. 10.1016/j.foodres.2017.06.028 28784519

[B31] LiCari J. J. (1970). *Salmonella* survival during spray drying and subsequent handling of skimmilk powder. II. Effects of drying conditions. *J. Dairy Sci.* 53 871–876. 10.3168/jds.S0022-0302(70)86309-3 4913266

[B32] Machado S. G. Baglinière F. Marchand S. Van Coillie E. (2017). The biodiversity of the microbiota producing heat-resistant enzymes responsible for spoilage in processed bovine milk and dairy products. *Front. Microbiol.* 8:302. 10.3389/fmicb.2017.00302 28298906 PMC5331058

[B33] Mazas M. Lopez M. Martinez S. Bernardo A. Martin R. (1999a). Heat resistance of *Bacillus cereus* spores: effects of milk constituents and stabilizing additives. *J. Food Prot.* 62 410–413. 10419217 10.4315/0362-028x-62.4.410

[B34] Mazas M. Martínez S. López M. Alvarez A. B. Martin R. (1999b). Thermal inactivation of *Bacillus cereus* spores affected by the solutes used to control water activity of the heating medium. *Int. J. Food Microbiol.* 53 61–67. 10.1016/S0168-1605(99)00145-2 10598115

[B35] McHugh A. J. Feehily C. Hill C. Cotter P. D. (2017). Detection and enumeration of spore-forming bacteria in powdered dairy products. *Front. Microbiol.* 8:109. 10.3389/fmicb.2017.00109 28197144 PMC5281614

[B36] Mugele R. A. Evans H. D. (1951). Droplet size distribution in sprays. *Ind. Eng. Chem.* 43 1317–1324. 10.1021/ie50498a023

[B37] Neubeck M. Von Baur C. Krewinkel M. Stoeckel M. Kranz B. Stressler T. (2015). Biodiversity of refrigerated raw milk microbiota and their enzymatic spoilage potential. *Int. J. Food Microbiol.* 211 57–65. 10.1016/j.ijfoodmicro.2015.07.001 26173200

[B38] Nolasco Júnior J. Massaguer P. R. (2007). Thermal death kinetics of *B. stearothermophillus* spores in sugarcane must. *J. Food Process Eng.* 30 625–639. 10.1111/j.1745-4530.2007.00122.x

[B39] Pereira P. C. (2014). Milk nutritional composition and its role in human health. *Nutrition* 30 619–627. 10.1016/j.nut.2013.10.011 24800664

[B40] Pflug I. J. (1999). *Microbiology and Engineering of Sterilization Process* 10th Edn. Minneapolis, MN: University of Minnesota.

[B41] Pispan S. Hewitt C. J. Stapley A. G. F. (2013). Comparison of cell survival rates of *E. coli* K12 and *L. acidophilus* undergoing spray drying. *Food Bioprod. Process.* 91 362–369. 10.1016/j.fbp.2013.01.005

[B42] Reyes J. Bastias J. Gutierrez M. Rodriguez M. (2007). Prevalence of *Bacillus cereus* in dried milk products used by Chilean School Feeding Program. *Food Microbiol.* 24 1–6. 10.1016/j.fm.2006.04.004 16943088

[B43] Sant’Ana A. S. Rosenthal A. Massaguer P. R. (2009). Heat resistance and the effects of continuous pasteurization on the inactivation of *Byssochlamys fulva* ascospores in clarified apple juice. *J. Appl. Microbiol.* 107 197–209. 10.1111/j.1365-2672.2009.04195.x 19298507

[B44] Santillana Farakos S. M. Schaffner D. W. Frank J. F. (2014). Predicting survival of *Salmonella* in low–water activity foods: an analysis of literature data. *J. Food Prot.* 77 1448–1461. 10.4315/0362-028X.JFP-14-013 25198835

[B45] Sapru V. Labuza T. P. (1993). Temperature dependence of thermal inactivation rate constants of bacterial spores in a glassy state. *J. Ind. Microbiol.* 12 247–250. 10.1007/BF01584197

[B46] Schebor C. del Pilar Buera M. Chirife J. (1996). Glassy state in relation to the thermal inactivation of the enzyme invertase in amorphous dried matrices of trehalose, maltodextrin and PVP. *J. Food Eng.* 30 269–282. 10.1016/S0260-8774(96)00058-1

[B47] Schuck P. (2002). Spray drying of dairy products: state of the art. *Lait* 82 375–382. 10.1051/lait:2002017

[B48] Schutyser M. A. I. Perdana J. Boom R. M. (2012). Single droplet drying for optimal spray drying of enzymes and probiotics. *Trends Food Sci. Technol.* 27 73–82. 10.1016/j.tifs.2012.05.006

[B49] Smelt J. P. Bos A. P. Kort R. Brul S. (2008). Modelling the effect of sub(lethal) heat treatment of *Bacillus subtilis* spores on germination rate and outgrowth to exponentially growing vegetative cells. *Int. J. Food Microbiol.* 128 34–40. 10.1016/j.ijfoodmicro.2008.08.023 18926580

[B50] Smelt J. P. Brul S. (2014). Thermal inactivation of microorganisms. *Crit. Rev. Food Sci. Nutr.* 54 1371–1385. 10.1080/10408398.2011.637645 24564593

[B51] Sollohub K. Cal K. (2010). Spray drying technique: II. Current applications in pharmaceutical technology. *J. Pharm. Sci.* 99 587–597. 10.1002/jps.21963 19862804

[B52] Spanu C. (2016). “Sporeforming bacterial pathogens in ready-to-eat dairy products,” in *Food Hygiene and Toxicology in Ready-to-Eat Foods* ed. Kotzekidou P. (New York City, NY: Elsevier Inc.) 259–273. 10.1016/B978-0-12-801916-0.00015-7

[B53] Stoeckel M. Atamer Z. Hinrichs J. (2014). Thermal inactivation of *Bacillus cereus* spores in micellar casein concentrates-effect of protein content and pH development. *Dairy Sci. Technol.* 94 539–548. 10.1007/s13594-014-0178-1

[B54] Stoeckel M. Westermann A. C. Atamer Z. Hinrichs J. (2013). Thermal inactivation of *Bacillus cereus* spores in infant formula under shear conditions. *Dairy Sci. Technol.* 93 163–175. 10.1007/s13594-012-0101-6

[B55] Taylor T. M. Pérez K. L. (2015). “67. Microbiological media, reagents, and stains,” in *Compendium of Methods for the Microbiological Examination of Foods* eds Salfinger Y. Tortorello M. L. (Washington, DC: American Public Health Association). 10.2105/MBEF.0222.072

[B56] Tiburski J. H. Rosenthal A. Guyot S. Perrier-Cornet J.-M. Gervais P. (2014). Water distribution in bacterial spores: a key factor in heat resistance. *Food Biophys.* 9 10–19. 10.1007/s11483-013-9312-5

[B57] Tri P. N. Domenek S. Guinault A. Sollogoub C. (2013). Crystallization behavior of poly(lactide)/poly(β-hydroxybutyrate)/talc composites. *J. Appl. Polym. Sci.* 129 3355–3365. 10.1002/app.39056

[B58] Wang J. Huang S. Fu N. Jeantet R. Chen X. D. (2016). Thermal aggregation of calcium-fortified skim milk enhances probiotic protection during convective droplet drying. *J. Agric. Food Chem.* 64 6003–6010. 10.1021/acs.jafc.6b02205 27420726

[B59] Wypych G. (2000). *HandbooK of Fillers* 2nd Edn. Toronto, ON: ChemTec Publishing.

[B60] Xueyong Z. Jianping D. Jianbao G. Ziniu Y. (2008). Activity-loss characteristics of spores of *Bacillus thuringiensis* during spray drying. *Food Bioprod. Process.* 86 37–42. 10.1016/j.fbp.2007.10.017

[B61] Yuan D. Zhang M. Li Y. (2018). Effect of intrinsic and extrinsic factors on the adhesion ability of thermophilic bacilli isolated from milk powders in the Chinese market. *Int. Dairy J.* 80 1–7. 10.1016/j.idairyj.2017.12.011

[B62] Zoccal R. (2016). *Conjuntura Atual da Produção de Leite no Mundo. Balde Branco*. Available at: http://www.baldebranco.com.br/conjuntura-atual-da-producao-de-leite-no-mundo/

