# Corrigendum: Quantifying the Responses of Three *Bacillus cereus* Strains in Isothermal Conditions and During Spray Drying of Different Carrier Agents

**DOI:** 10.3389/fmicb.2018.02876

**Published:** 2018-11-28

**Authors:** Verônica O. Alvarenga, Fernanda B. Campagnollo, Arthur K. R. Pia, Deborah A. Conceição, Yuri Abud, Celso Sant'Anna, Miriam D. Hubinger, Anderson S. Sant'Ana

**Affiliations:** ^1^Department of Food Science, Faculty of Food Engineering, University of Campinas, Campinas, Brazil; ^2^Laboratory of Biotechnology (Labio), Metrology Applied to Life Science Division – National Institute of Metrology, Quality and Technology (Inmetro), Duque de Caxias, Brazil; ^3^Department of Food Engineering, Faculty of Food Engineering, University of Campinas, Campinas, Brazil

**Keywords:** spores, low water activity, thermal processing, dried foods, sporeforming bacteria, dairy products, milk, foodborne pathogens

In the original article, we neglected to include the funder Fundação de Amparo à Pesquisa do Estado de São Paulo (FAPESP), 18/09442-0 to AS. The authors apologize for this error and state that this does not change the scientific conclusions of the article in any way. The original article has been updated.

## Conflict of interest statement

The authors declare that the research was conducted in the absence of any commercial or financial relationships that could be construed as a potential conflict of interest.

